# *N*-[4-(4-Fluoro­phen­yl)-5-hy­droxy­methyl-6-isopropyl­pyrimidin-2-yl]-*N*-methyl­methane­sulfonamide

**DOI:** 10.1107/S1600536811049051

**Published:** 2011-11-25

**Authors:** Hong Xu, Hong-shun Sun, Feng-mao Luo

**Affiliations:** a Department of Chemical Engineering, Nanjing College of Chemical Technology, Geguan Road No. 265 Nanjing, Nanjing 210048, People’s Republic of China; b Department of Applied Chemistry, Nanjing College of Chemical Technology, Geguan Road No. 265 Nanjing, Nanjing 210048, People’s Republic of China; c Nanjing Xiansheng Dongyuan Pharmaceutic Company Limited, Xinglong Road No. 8 Nanjing, Nanjing 211800, People’s Republic of China

## Abstract

In the title compound, C_16_H_20_FN_3_O_3_S, the pyrimidine and benzene rings are oriented at a dihedral angle of 38.8 (3)°. An intra­molecular C—H⋯O hydrogen bond occurs. The crystal structure is stabilized by O—H⋯N hydrogen bonds. In addition, C—H⋯O inter­actions are also present.

## Related literature

For a related structure, see: He *et al.* (2008 ▶).
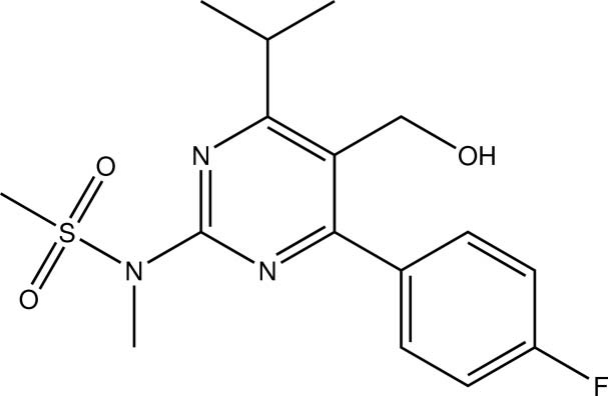


## Experimental

### 

#### Crystal data

C_16_H_20_FN_3_O_3_S
*M* *_r_* = 353.41
Monoclinic, 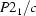

*a* = 5.8080 (12) Å
*b* = 11.803 (2) Å
*c* = 25.867 (5) Å
β = 93.10 (3)°
*V* = 1770.6 (6) Å^3^
*Z* = 4
Mo *K*α radiation
μ = 0.21 mm^−1^
*T* = 293 K
0.30 × 0.20 × 0.10 mm

#### Data collection

Enraf–Nonius CAD-4 diffractometer
Absorption correction: ψ scan (North *et al.*, 1968 ▶) *T*_min_ = 0.939, *T*_max_ = 0.979
3576 measured reflections
3234 independent reflections
2200 reflections with *I* > 2σ(*I*)
*R*_int_ = 0.034
3 standard reflections every 200 reflections  intensity decay: 1%

#### Refinement

*R*[*F* ^2^ > 2σ(*F* ^2^)] = 0.057
*wR*(*F* ^2^) = 0.168
*S* = 1.00
3234 reflections
217 parameters
H-atom parameters constrained
Δρ_max_ = 0.27 e Å^−3^
Δρ_min_ = −0.35 e Å^−3^

### 

Data collection: *CAD-4 EXPRESS* (Enraf–Nonius, 1994 ▶); cell refinement: *CAD-4 EXPRESS*; data reduction: *XCAD4* (Harms & Wocadlo, 1995 ▶); program(s) used to solve structure: *SHELXS97* (Sheldrick, 2008 ▶); program(s) used to refine structure: *SHELXL97* (Sheldrick, 2008 ▶); molecular graphics: *SHELXTL* (Sheldrick, 2008 ▶); software used to prepare material for publication: *SHELXL97*.

## Supplementary Material

Crystal structure: contains datablock(s) I, global. DOI: 10.1107/S1600536811049051/pv2454sup1.cif

Structure factors: contains datablock(s) I. DOI: 10.1107/S1600536811049051/pv2454Isup2.hkl

Supplementary material file. DOI: 10.1107/S1600536811049051/pv2454Isup3.cml

Additional supplementary materials:  crystallographic information; 3D view; checkCIF report

## Figures and Tables

**Table 1:** Hydrogen-bond geometry (Å, °)

*D*—H⋯*A*	*D*—H	H⋯*A*	*D*⋯*A*	*D*—H⋯*A*
O1—H1*A*⋯N2^i^	0.82	2.10	2.896 (3)	165
C5—H5*A*⋯O1	0.93	2.53	3.309 (4)	141
C15—H15*C*⋯O3^ii^	0.96	2.39	3.350 (4)	176
C16—H16*A*⋯O3^iii^	0.96	2.55	3.402 (4)	148

Symmetry codes: (i) 
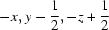
; (ii) 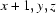; (iii) 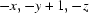.

**Fig. 1.**
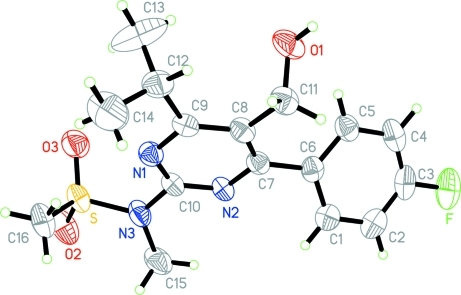
The molecular structure of the title compound showing displacement ellipsoids at the 50% probability level.

## supplementary crystallographic information

### Comment

The derivatives of pyrimidine are important chemical compound. We report here the crystal structure of the title compound.

In the title molecule (Fig. 1), the pyrimidine (N1/N2/C7—C10) and benzene (C1—C6) rings are inclined at a dihedral angle of 38.7 (3)°. The structure is stabilized by intermolecular hydrogen bonding of the type O—H···N. In addition, intramolecular C—H···N and C—H···O and intermolecular C—H···O hydrogen bonding interactions are also present in the crystal structure (Table 1). The crystal structure of a related compound has been reported recently (He *et al.*, 2008).

### Experimental

For the preparation of the title compound, sodium salt of *N*-methyl methane sulphonamide (106 g, 631.00 mmol) and 4-(4-fluorophenyl)-6-isopropyl- 2-methyl sulfonylpyrimidine-5-methanol (92 g, 284.0 mmol) were added to dimethylformamide (1000 ml) in a round bottom flask, and then stirred for 1 h at 303 K. After completion of the reaction, demineralized water (1000 ml) was added and stirred for 1 h. The mixture was filtered, washed with water, and then dried (yield; 90%). Crystals of the title compound suitable for X-ray analysis were obtained by slow evaporation of a methanol solution.

### Refinement

H atoms were positioned geometrically with O—H = 0.82 Å and C—H = 0.93, 0.96, 0.97 and 0.98 Å for aryl, methyl, methylene and methine H atoms, respectively, and constrained to ride on their parent atoms with *U*_iso_(H) = 1.5 times *U*_eq_(C-methyl), and 1.2 times *U*_eq_(all other C/O atoms).

### Crystal data

**Table:**

C_16_H_20_FN_3_O_3_S	*F*(000) = 744
*M**_r_* = 353.41	*D*_x_ = 1.326 Mg m^−^^3^
Monoclinic, *P*2_1_/*c*	Mo *K*α radiation, λ = 0.71073 Å
Hall symbol: -P 2ybc	Cell parameters from 25 reflections
*a* = 5.8080 (12) Å	θ = 9–13°
*b* = 11.803 (2) Å	µ = 0.21 mm^−^^1^
*c* = 25.867 (5) Å	*T* = 293 K
β = 93.10 (3)°	Block, colorless
*V* = 1770.6 (6) Å^3^	0.30 × 0.20 × 0.10 mm
*Z* = 4	

### Data collection

**Table:**

Enraf–Nonius CAD-4 diffractometer	2200 reflections with *I* > 2σ(*I*)
Radiation source: fine-focus sealed tube	*R*_int_ = 0.034
graphite	θ_max_ = 25.4°, θ_min_ = 1.6°
ω/2θ scans	*h* = 0→7
Absorption correction: ψ scan (North *et al.*, 1968)	*k* = 0→14
*T*_min_ = 0.939, *T*_max_ = 0.979	*l* = −31→31
3576 measured reflections	3 standard reflections every 200 reflections
3234 independent reflections	intensity decay: 1%

### Refinement

**Table:**

Refinement on *F*^2^	Primary atom site location: structure-invariant direct methods
Least-squares matrix: full	Secondary atom site location: difference Fourier map
*R*[*F*^2^ > 2σ(*F*^2^)] = 0.057	Hydrogen site location: inferred from neighbouring sites
*wR*(*F*^2^) = 0.168	H-atom parameters constrained
*S* = 1.00	*w* = 1/[σ^2^(*F*_o_^2^) + (0.098*P*)^2^] where *P* = (*F*_o_^2^ + 2*F*_c_^2^)/3
3234 reflections	(Δ/σ)_max_ < 0.001
217 parameters	Δρ_max_ = 0.27 e Å^−^^3^
0 restraints	Δρ_min_ = −0.35 e Å^−^^3^

### Special details

**Table:**

Geometry. All e.s.d.'s (except the e.s.d. in the dihedral angle between two l.s. planes) are estimated using the full covariance matrix. The cell e.s.d.'s are taken into account individually in the estimation of e.s.d.'s in distances, angles and torsion angles; correlations between e.s.d.'s in cell parameters are only used when they are defined by crystal symmetry. An approximate (isotropic) treatment of cell e.s.d.'s is used for estimating e.s.d.'s involving l.s. planes.
Refinement. Refinement of *F*^2^ against ALL reflections. The weighted *R*-factor *wR* and goodness of fit *S* are based on *F*^2^, conventional *R*-factors *R* are based on *F*, with *F* set to zero for negative *F*^2^. The threshold expression of *F*^2^ > σ(*F*^2^) is used only for calculating *R*-factors(gt) *etc*. and is not relevant to the choice of reflections for refinement. *R*-factors based on *F*^2^ are statistically about twice as large as those based on *F*, and *R*- factors based on ALL data will be even larger.

### Fractional atomic coordinates and isotropic or equivalent isotropic displacement parameters (Å^2^)

**Table:**

	*x*	*y*	*z*	*U*_iso_*/*U*_eq_	
S	0.19836 (15)	0.47414 (7)	0.06975 (3)	0.0468 (3)	
F	0.3816 (4)	0.31374 (19)	0.44364 (7)	0.0810 (7)	
O1	−0.2240 (4)	0.06223 (19)	0.24443 (9)	0.0574 (7)	
H1A	−0.2602	0.0043	0.2595	0.086*	
N1	0.1043 (4)	0.2659 (2)	0.12672 (9)	0.0390 (6)	
C1	0.4508 (6)	0.3093 (3)	0.30694 (13)	0.0491 (8)	
H1C	0.5589	0.3309	0.2836	0.059*	
O2	0.2945 (5)	0.58313 (19)	0.06090 (9)	0.0665 (7)	
N2	0.2636 (4)	0.34440 (19)	0.20528 (9)	0.0358 (6)	
C2	0.4998 (7)	0.3252 (3)	0.35873 (14)	0.0572 (9)	
H2C	0.6402	0.3558	0.3708	0.069*	
N3	0.3087 (5)	0.4355 (2)	0.12759 (9)	0.0454 (7)	
O3	−0.0463 (4)	0.4631 (2)	0.06957 (9)	0.0610 (7)	
C3	0.3365 (7)	0.2948 (3)	0.39237 (12)	0.0548 (9)	
C4	0.1316 (7)	0.2479 (3)	0.37660 (13)	0.0550 (9)	
H4A	0.0251	0.2278	0.4006	0.066*	
C5	0.0833 (6)	0.2305 (3)	0.32381 (12)	0.0453 (8)	
H5A	−0.0562	0.1979	0.3124	0.054*	
C6	0.2421 (5)	0.2614 (2)	0.28818 (11)	0.0384 (7)	
C7	0.1927 (5)	0.2532 (2)	0.23138 (11)	0.0362 (7)	
C8	0.0778 (5)	0.1639 (2)	0.20606 (11)	0.0383 (7)	
C9	0.0296 (5)	0.1763 (2)	0.15288 (11)	0.0374 (7)	
C10	0.2204 (5)	0.3436 (2)	0.15435 (11)	0.0358 (7)	
C11	0.0129 (6)	0.0571 (3)	0.23370 (13)	0.0461 (8)	
H11A	0.0408	−0.0083	0.2122	0.055*	
H11B	0.1062	0.0496	0.2658	0.055*	
C12	−0.1153 (6)	0.0935 (3)	0.12057 (13)	0.0510 (9)	
H12A	−0.1274	0.0234	0.1405	0.061*	
C13	−0.3530 (8)	0.1421 (5)	0.1122 (2)	0.117 (2)	
H13A	−0.4160	0.1570	0.1450	0.176*	
H13B	−0.4498	0.0889	0.0932	0.176*	
H13C	−0.3455	0.2114	0.0929	0.176*	
C14	−0.0079 (9)	0.0648 (4)	0.07052 (16)	0.0964 (17)	
H14A	0.1431	0.0337	0.0778	0.145*	
H14B	0.0039	0.1322	0.0500	0.145*	
H14C	−0.1023	0.0102	0.0518	0.145*	
C15	0.5194 (6)	0.4925 (3)	0.14861 (13)	0.0599 (10)	
H15A	0.5627	0.4619	0.1821	0.090*	
H15B	0.4908	0.5722	0.1516	0.090*	
H15C	0.6419	0.4803	0.1258	0.090*	
C16	0.3091 (7)	0.3781 (3)	0.02622 (12)	0.0582 (10)	
H16A	0.2511	0.3961	−0.0083	0.087*	
H16B	0.2629	0.3027	0.0349	0.087*	
H16C	0.4744	0.3828	0.0281	0.087*	

### Atomic displacement parameters (Å^2^)

**Table:**

	*U*^11^	*U*^22^	*U*^33^	*U*^12^	*U*^13^	*U*^23^
S	0.0611 (6)	0.0414 (5)	0.0392 (5)	0.0019 (4)	0.0142 (4)	0.0066 (3)
F	0.124 (2)	0.0785 (16)	0.0398 (12)	0.0170 (14)	−0.0057 (12)	−0.0016 (10)
O1	0.0590 (16)	0.0441 (13)	0.0707 (16)	−0.0090 (11)	0.0171 (12)	0.0151 (11)
N1	0.0419 (14)	0.0378 (14)	0.0376 (14)	−0.0066 (12)	0.0051 (11)	−0.0017 (11)
C1	0.048 (2)	0.052 (2)	0.0471 (19)	0.0018 (16)	0.0007 (15)	0.0042 (15)
O2	0.099 (2)	0.0409 (14)	0.0616 (16)	−0.0055 (13)	0.0266 (14)	0.0134 (11)
N2	0.0414 (14)	0.0336 (13)	0.0332 (13)	−0.0017 (11)	0.0096 (10)	0.0011 (10)
C2	0.061 (2)	0.056 (2)	0.054 (2)	0.0067 (18)	−0.0092 (18)	0.0006 (17)
N3	0.0580 (17)	0.0442 (15)	0.0345 (14)	−0.0159 (13)	0.0089 (12)	0.0010 (11)
O3	0.0540 (15)	0.0709 (17)	0.0590 (15)	0.0089 (12)	0.0095 (11)	0.0162 (12)
C3	0.084 (3)	0.0451 (19)	0.0352 (18)	0.0163 (19)	−0.0012 (18)	0.0034 (15)
C4	0.077 (3)	0.047 (2)	0.0422 (19)	0.0133 (19)	0.0180 (18)	0.0092 (15)
C5	0.0510 (19)	0.0401 (17)	0.0455 (18)	0.0035 (15)	0.0077 (15)	0.0080 (14)
C6	0.0438 (17)	0.0320 (15)	0.0394 (17)	0.0104 (14)	0.0034 (13)	0.0044 (12)
C7	0.0348 (16)	0.0353 (16)	0.0392 (16)	0.0049 (13)	0.0079 (12)	0.0038 (13)
C8	0.0417 (17)	0.0310 (15)	0.0428 (17)	0.0009 (13)	0.0079 (13)	0.0027 (13)
C9	0.0343 (16)	0.0345 (16)	0.0442 (17)	−0.0026 (13)	0.0089 (13)	−0.0017 (13)
C10	0.0387 (17)	0.0346 (16)	0.0349 (16)	−0.0016 (13)	0.0104 (12)	0.0001 (12)
C11	0.050 (2)	0.0355 (17)	0.054 (2)	0.0001 (14)	0.0067 (15)	0.0064 (14)
C12	0.051 (2)	0.0440 (19)	0.058 (2)	−0.0140 (16)	0.0015 (16)	−0.0028 (15)
C13	0.055 (3)	0.106 (4)	0.186 (6)	0.000 (3)	−0.040 (3)	−0.042 (4)
C14	0.109 (4)	0.110 (4)	0.073 (3)	−0.052 (3)	0.026 (3)	−0.047 (3)
C15	0.065 (2)	0.062 (2)	0.054 (2)	−0.0297 (19)	0.0150 (17)	−0.0032 (17)
C16	0.080 (3)	0.058 (2)	0.0375 (18)	0.008 (2)	0.0176 (17)	0.0002 (16)

### Geometric parameters (Å, °)

**Table:**

S—O2	1.426 (2)	C6—C7	1.485 (4)
S—O3	1.427 (3)	C7—C8	1.391 (4)
S—N3	1.659 (3)	C8—C9	1.396 (4)
S—C16	1.745 (3)	C8—C11	1.507 (4)
F—C3	1.356 (4)	C9—C12	1.511 (4)
O1—C11	1.419 (4)	C11—H11A	0.9700
O1—H1A	0.8200	C11—H11B	0.9700
N1—C10	1.325 (4)	C12—C13	1.500 (6)
N1—C9	1.340 (4)	C12—C14	1.506 (5)
C1—C2	1.367 (4)	C12—H12A	0.9800
C1—C6	1.400 (4)	C13—H13A	0.9600
C1—H1C	0.9300	C13—H13B	0.9600
N2—C10	1.328 (3)	C13—H13C	0.9600
N2—C7	1.347 (4)	C14—H14A	0.9600
C2—C3	1.369 (5)	C14—H14B	0.9600
C2—H2C	0.9300	C14—H14C	0.9600
N3—C10	1.399 (4)	C15—H15A	0.9600
N3—C15	1.474 (4)	C15—H15B	0.9600
C3—C4	1.355 (5)	C15—H15C	0.9600
C4—C5	1.395 (4)	C16—H16A	0.9600
C4—H4A	0.9300	C16—H16B	0.9600
C5—C6	1.388 (4)	C16—H16C	0.9600
C5—H5A	0.9300		
			
O2—S—O3	118.72 (16)	N1—C10—N2	127.1 (3)
O2—S—N3	104.90 (14)	N1—C10—N3	117.4 (3)
O3—S—N3	108.28 (14)	N2—C10—N3	115.5 (2)
O2—S—C16	108.74 (16)	O1—C11—C8	109.1 (2)
O3—S—C16	109.90 (18)	O1—C11—H11A	109.9
N3—S—C16	105.41 (16)	C8—C11—H11A	109.9
C11—O1—H1A	109.5	O1—C11—H11B	109.9
C10—N1—C9	116.3 (2)	C8—C11—H11B	109.9
C2—C1—C6	121.7 (3)	H11A—C11—H11B	108.3
C2—C1—H1C	119.2	C13—C12—C14	112.5 (4)
C6—C1—H1C	119.2	C13—C12—C9	108.3 (3)
C10—N2—C7	116.5 (2)	C14—C12—C9	112.1 (3)
C1—C2—C3	118.2 (3)	C13—C12—H12A	107.9
C1—C2—H2C	120.9	C14—C12—H12A	107.9
C3—C2—H2C	120.9	C9—C12—H12A	107.9
C10—N3—C15	119.3 (3)	C12—C13—H13A	109.5
C10—N3—S	121.7 (2)	C12—C13—H13B	109.5
C15—N3—S	118.7 (2)	H13A—C13—H13B	109.5
C4—C3—F	118.8 (3)	C12—C13—H13C	109.5
C4—C3—C2	122.9 (3)	H13A—C13—H13C	109.5
F—C3—C2	118.3 (4)	H13B—C13—H13C	109.5
C3—C4—C5	118.8 (3)	C12—C14—H14A	109.5
C3—C4—H4A	120.6	C12—C14—H14B	109.5
C5—C4—H4A	120.6	H14A—C14—H14B	109.5
C6—C5—C4	120.4 (3)	C12—C14—H14C	109.5
C6—C5—H5A	119.8	H14A—C14—H14C	109.5
C4—C5—H5A	119.8	H14B—C14—H14C	109.5
C5—C6—C1	118.1 (3)	N3—C15—H15A	109.5
C5—C6—C7	122.7 (3)	N3—C15—H15B	109.5
C1—C6—C7	119.1 (3)	H15A—C15—H15B	109.5
N2—C7—C8	121.4 (3)	N3—C15—H15C	109.5
N2—C7—C6	113.3 (3)	H15A—C15—H15C	109.5
C8—C7—C6	125.3 (3)	H15B—C15—H15C	109.5
C7—C8—C9	116.8 (3)	S—C16—H16A	109.5
C7—C8—C11	122.4 (3)	S—C16—H16B	109.5
C9—C8—C11	120.8 (3)	H16A—C16—H16B	109.5
N1—C9—C8	121.8 (3)	S—C16—H16C	109.5
N1—C9—C12	114.6 (3)	H16A—C16—H16C	109.5
C8—C9—C12	123.6 (3)	H16B—C16—H16C	109.5
			
C6—C1—C2—C3	−1.2 (5)	C6—C7—C8—C9	174.4 (3)
O2—S—N3—C10	167.0 (2)	N2—C7—C8—C11	175.2 (3)
O3—S—N3—C10	39.2 (3)	C6—C7—C8—C11	−7.2 (4)
C16—S—N3—C10	−78.3 (3)	C10—N1—C9—C8	−1.9 (4)
O2—S—N3—C15	−19.4 (3)	C10—N1—C9—C12	176.2 (3)
O3—S—N3—C15	−147.1 (3)	C7—C8—C9—N1	4.6 (4)
C16—S—N3—C15	95.3 (3)	C11—C8—C9—N1	−173.8 (3)
C1—C2—C3—C4	1.3 (5)	C7—C8—C9—C12	−173.3 (3)
C1—C2—C3—F	−178.0 (3)	C11—C8—C9—C12	8.3 (4)
F—C3—C4—C5	178.8 (3)	C9—N1—C10—N2	−2.6 (4)
C2—C3—C4—C5	−0.5 (5)	C9—N1—C10—N3	177.3 (2)
C3—C4—C5—C6	−0.4 (5)	C7—N2—C10—N1	4.0 (4)
C4—C5—C6—C1	0.5 (4)	C7—N2—C10—N3	−175.9 (2)
C4—C5—C6—C7	−175.1 (3)	C15—N3—C10—N1	−151.1 (3)
C2—C1—C6—C5	0.3 (5)	S—N3—C10—N1	22.6 (4)
C2—C1—C6—C7	176.1 (3)	C15—N3—C10—N2	28.9 (4)
C10—N2—C7—C8	−0.8 (4)	S—N3—C10—N2	−157.5 (2)
C10—N2—C7—C6	−178.7 (2)	C7—C8—C11—O1	100.5 (3)
C5—C6—C7—N2	136.6 (3)	C9—C8—C11—O1	−81.2 (3)
C1—C6—C7—N2	−39.0 (4)	N1—C9—C12—C13	−77.6 (4)
C5—C6—C7—C8	−41.2 (4)	C8—C9—C12—C13	100.4 (4)
C1—C6—C7—C8	143.3 (3)	N1—C9—C12—C14	47.0 (4)
N2—C7—C8—C9	−3.1 (4)	C8—C9—C12—C14	−134.9 (4)

### Hydrogen-bond geometry (Å, °)

**Table:**

*D*—H···*A*	*D*—H	H···*A*	*D*···*A*	*D*—H···*A*
O1—H1A···N2^i^	0.82	2.10	2.896 (3)	165.
C5—H5A···O1	0.93	2.53	3.309 (4)	141.
C15—H15A···N2	0.96	2.33	2.765 (4)	107.
C15—H15C···O3^ii^	0.96	2.39	3.350 (4)	176.
C16—H16A···O3^iii^	0.96	2.55	3.402 (4)	148.

Symmetry codes: (i) −*x*, *y*−1/2, −*z*+1/2; (ii) *x*+1, *y*, *z*; (iii) −*x*, −*y*+1, −*z*.
